# Wildlife corridors in a Southern African conservation landscape: the political ecology of multispecies mobilities along the arteries of anthropogenic conservation

**DOI:** 10.1080/23323256.2024.2327467

**Published:** 2024-08-08

**Authors:** Michael Bollig

**Affiliations:** Environmental Anthropology, University of Cologne, Cologne, Germany

**Keywords:** coexistence landscape, conservation, elephants, land-use change, wildlife corridors

## Abstract

The decline of biodiversity is a key topic in public discussions around the globe. These debates have triggered massive efforts to increase protected areas and to safeguard the corridors connecting them. The wildlife corridors dealt with in this article are mainly thought to facilitate the mobility of elephants and some other large herbivores (for example, zebra and buffalo). Wildlife corridors are not only essential for species connectivity but also an integral part of the booming ecotourism in north-eastern Namibia’s conservation landscapes. Coexistence infrastructure is meant to contribute to economic development and local incomes. Conservancies — community-based conservation organisations in the Namibian context — gazette corridors and market wildlife abundance to ecotourists, potential investors in tourism and commercial hunters. The coexistence of humans and wildlife is challenging, though. Human-wildlife interactions frequently result in damage, and often conservationist environmental infrastructuring runs against the aims of farmers to expand their fields for commercial crop production and to gain pastures for growing cattle herds. It also runs against other governmentally endorsed infrastructuring that brings tarred roads, water pipelines and boreholes. This article analyses contested wildlife corridors as part of a larger conservationist project in the western parts of Namibia’s Zambezi Region.

## Abstract (pt)

O declínio da biodiversidade é uma questão chave em discussões públicas em todo o mundo. Estes debates desencadearam esforços massivos para aumentar as áreas protegidas e para salvaguardar os corredores que as conectam. Os corredores de vida selvagem tratados neste artigo são pensados principalmente para facilitar a mobilidade de elefantes e de alguns outros grandes herbívoros (por exemplo, zebras e búfalos). Os corredores de vida selvagem não são apenas essenciais para a conexão das espécies, mas também como uma parte integrante do ecoturismo em expansão nas paisagens de conservação do nordeste da Namíbia. A infraestrutura de coexistência destina-se a contribuir para o desenvolvimento econômico e para os rendimentos locais. As conservações — organizações de conservação de base comunitária no contexto da Namíbia — anunciam os corredores e comercializam a abundância da vida selvagem para ecoturistas, potenciais investidores no turismo e caçadores comerciais. Contudo, a coexistência de humanos e vida selvagem é desafiadora. As interações entre os humanos e a vida selvagem frequentemente resultam em danos, e muitas vezes a infraestrutura ambiental conservacionista vai contra os objetivos dos fazendeiros de expandir suas terras para a produção agrícola comercial e de obter pastagens para os crescentes rebanhos de gado. Também vai contra outros projetos de infraestrutura apoiados pelo governo que trazem estradas asfaltadas, tubulações de água e poços. Este artigo analisa os controversos corredores de vida selvagem como parte de um projeto conservacionista mais amplo nas partes ocidentais da região do Zambeze na Namíbia.

## Introduction

The massive decline of biodiversity is a key topic in public discussions around the globe. The causes and consequences of species extinctions and population declines are being analysed in the world’s top natural science journals (see, for example, Ceballos, Ehrlich and Dirzo 2017), depicted in monograph length in popular science volumes (for example, Wilson 2016) and dramatically staged in exhibitions, artwork and literature. These reports and projections have triggered substantial efforts to increase protected areas on a global scale and to safeguard the corridors connecting them. Protected landscapes and what are technically called other effective area-based conservation measures are often addressed as coexistence landscapes. Linked to the concept of coexistence are imageries, narratives and practices to create and/or maintain an environmental infrastructure consisting of core conservation zones, buffer zones and corridors that are conducive to ecological connectivity. Humans and wildlife together are the architects of such coexistence in general and of corridors used by wildlife in particular. Such corridors of connectivity in coexistence landscapes habitually prioritise the needs of some species deemed to be particularly valuable or threatened. The wildlife corridors dealt with in this contribution are mainly thought to facilitate the mobility of elephants and some other large herbivores (for example, zebra and buffalo). They are less salient for carnivores, hippos or smaller antelopes, let alone birds, fish or reptiles.

There are several excellent contributions about human-elephant engagements in the Asian context, where humans interact with tamed elephants and only sometimes with wild elephants. The contributions by Locke (2013), Barua (2014) and Lorimer (2010) depict elephants as sensitive and sociable beings with a complex species history and an astonishingly complex communicative repertoire. For the African context, such a multispecies perspective on elephants is still lacking. This article contributes to an emergent African multispecies perspective on elephants. In Africa, elephants are not companions, as Lorimer (2010) carefully describes for the Sri Lankan and Münster (2016) for the southern Indian context. Often they are competitors, and sometimes foes that raid crop fields and occasionally harm and kill people. However, to reduce human-elephant relations to conflict would not do justice to the complexity of these relations (Mumby and Plotnik 2018). Informants I had the chance to talk to in north-eastern Namibia habitually had deep respect for elephants, displayed an intimate knowledge of elephant sensing, sociality and memory and theorised about the elephant mind, their superior smelling sense and their approach to place making. Elephants were not companions, but well known, respected and occasionally feared nearby neighbours sharing a landscape with humans.

Wildlife corridors are not only essential for species connectivity but are also an integral part of the booming ecotourism in north-eastern Namibia’s conservation landscapes. Coexistence infrastructure is meant to contribute to economic development and local incomes. Lodges and camping grounds line up in the vicinity of wildlife corridors and at the border of national parks. Conservancies — community-based conservation organisations in the Namibian context — gazette corridors and market wildlife abundance to ecotourists, potential investors in tourism and commercial hunters (Kalvelage, Revilla Diez and Bollig 2020). NGOs and donor organisations have developed programmes (for example, the Wildlife Credit Program by the World Wide Fund for Nature) to foster the continued existence of corridors that are regarded as absolutely essential for the success of wildlife conservation. With Remis and Jost Robinson (2020, 463), who comment on a central African elephant corridor, I argue that wildlife corridors represent places “where the past, present and future lives of elephants and other wildlife, human communities, global and local economies, conservation, research, [and] tourism … converge.”

The coexistence of humans and wildlife is challenging, though. Human-wildlife interactions frequently result in damage, and often conservationist environmental infrastructuring runs against the aims of farmers to expand their fields for commercial crop production and to gain pastures for growing cattle herds. It also conflicts occasionally with other governmentally endorsed infrastructuring that brings tarred roads, water pipelines and boreholes.

This article analyses contested wildlife corridors as part of a larger conservationist project in the western parts of Namibia’s Zambezi Region, the so-called Mudumu Landscape (named after a national park in that area). This landscape is a key part of the giant Kavango-Zambezi Transfrontier Conservation Area (KAZA TFCA), founded in 2011, and at c. 520 000 km^2^ the world’s largest conservation area (Dittmann and Müller-Mahn 2023). The Mudumu Landscape falls into separate categories of conservation: three national parks of smaller size (Nkasa Rupara, Mudumu and Bwabwata), community-based conservancies and community forests. In recent decades, wildlife numbers in Namibia’s Mudumu Landscape have increased significantly. Particularly elephant numbers have risen to such a degree that confrontations between humans and elephants are becoming continuously more frequent during some periods of the year. The Mudumu Landscape also has a sizeable number of tourist establishments, notably lodges and camping grounds (Kalvelage, Revilla Diez and Bollig 2020). Such tourist infrastructure facilitates the diversification of rural livelihoods and allows for non-agricultural income, specifically from ecotourism (Mayer et al. 2022) and trophy hunting (Kalvelage, Revilla Diez and Bollig 2023). At the same time, expanding agricultural production and cattle husbandry claim space (Bollig and Vehrs 2020), and it is especially hitherto little-used stretches of land that come into focus. These are often the same stretches of land that have been endorsed by conservancies, non-governmental organisations (NGOs) and the administration as wildlife corridors.

Based on theoretical explorations on environmental infrastructures (Bollig and Krause 2023, 67–78), political ecology (Stott and Sullivan 2000) and multispecies anthropology (Locke and Muenster 2015), this article sets out to capture the complex engagements within multispecies assemblages (Lacan et al. forthcoming) in and along wildlife corridors. I regard wildlife corridors as environmental infrastructure co-constituted by wildlife agency as much as by human agency. I specifically emphasise that competing projects of environmental infrastructuring are under way, simultaneously shaped by various political and economic agendas and the demographic and social dynamics of wildlife species. The contribution is informed by a political-ecology perspective as it analyses how power and wealth lead to specific claims towards land and peculiar practices of land governance. The article is obliged to adopt a multispecies perspective in that it attempts to take into account the sociality, demographic history, sensing and communicative repertoires, especially of elephants (for a fascinating zoological account of these aspects, see Garstang 2015). Multispecies approaches suggest that mutual understanding between humans and elephants is possible (Locke 2013). Many of my informants would suggest that by virtue of prior knowledge and attentiveness, humans can understand elephant agency to a significant extent. At the same time, they allege that elephants also have some understanding of humans, differentiate their smells and remember activities directed at them.

In the subsequent sections, I first highlight a few key concepts that are constitutive of contemporary conservationist imageries and narratives. This includes briefly laying out my key analytical concept: environmental infrastructure. I then describe how wildlife corridors came to be defined in the Mudumu Landscape and how they were meant to link core conservation areas across boundaries. I present the biographies of three separate corridors and show that contestations and co-adaptations differ from place to place. The third section details the contestations wildlife corridors are undergoing in the present. Rapidly expanding farming activities and increasing cattle herds claim the same space that has been earmarked for wildlife corridors. At the same time, major changes in settlement patterns are observable as what used to be consolidated villages disintegrate into vast linear settlements. Conservationist organisations, national and international, the staff of respective line ministries and the committees of community-based conservancies work against the rapid demise of wildlife corridors as the key conservation concern of the KAZA TFCA. But it is not only challenges brought about by farming that are discussed. The rapid increase of wildlife numbers in the region has certainly contributed to an escalation of these challenges. It is especially the increase in elephant numbers and many incidences of direct human-elephant contact that provide the empirical and experiential basis for local in-depth observations on elephant sensing, sociality and spatial behaviour. The fourth section discusses the programmes and projects to further wildlife corridors as key infrastructure of a conservation landscape. In the concluding part, I summarise my results and outline options for a compromise. I also highlight what an approach that combines insights of the anthropology of infrastructure and of multispecies approaches may offer to conservationist challenges in a coexistence landscape.

The article is based on empirical fieldwork in the Mudumu Landscape over many months between 2018 and 2023. I specifically focused on wildlife corridors in three conservancies (Wuparo and Balyerwa in Mudumu South and Mashi in Mudumu North) where I did numerous interviews with small-scale farmers, game guards and other staff of community conservancies, NGO employees in charge of wildlife corridor management and administrative staff in charge of wildlife conservation. I also conducted a good number of interviews on human insights into elephant behaviour. Further, I conducted interviews with experts in the urban capital of the region, Katima Mulilo. The study profited from ample access to planning data and data recorded by game guards on human-wildlife conflicts, wildlife demographics and conservation-related economies. During the research, I was not linked to any NGO or government body, but I certainly benefitted from long-term good relations with both (I have been doing research in Namibia since the mid-1990s). Whilst doing research, I lived in both the Mashi and Wuparo Conservancies, and this is also where most of my insights on human-elephant relations developed.

## Key concepts: connectivity, corridors and coexistence

Since the 1990s, “connectivity” has become the key term in large-scale conservation efforts, a shift aptly summarised by eco-journalist Fraser (2009, 27) who proclaims that connectivity “took over the conservation world the way the iPod took over music” in the 1990s. Connectivity is defined as “the extent to which a species or population can move among landscape elements in a mosaic of habitat types” (Hilty, Lidicker and Merenlender 2006, 90). Indeed, since the Rio de Janeiro Earth Summit in 1992, connectivity has become an ingredient in all conservation planning, and all major conservation agencies have made connectivity planning a key concern. Corridors were the environmental infrastructure that facilitated connectivity (Hilty, Lidicker and Merenlender 2006, 89). Technically, corridors are defined as “linear landscape elements that connect two or more patches of natural habitat and function to facilitate movement”, and “any space identifiable by species using it that facilitates the movement of animals or plants over time between two or more patches of otherwise disjunct habitat” (Hilty, Lidicker and Merenlender 2006, 90). The Wildlife Corridor Policy for Namibia’s Zambezi Region discusses wildlife corridors in this way (GoN 2022).

A multispecies perspective suggests yet another view of connectivity and corridors. Connectivity means the capacity to exchange information and to find mates and companions across distances. It means the capacity to roam around and gain experience in a larger spatial setting. Corridors are paths that prior generations of a species have moved through. They have changed the earth and vegetation on these paths profoundly. Garstang (2015, 10) reports that “the surface of these trails is often a mixture of sand and fibre from centuries of droppings, producing a soft, spongy surface that can be trodden in silence.” It is not exactly known why elephants use some paths, apparently across generations, but it is certain that they have an excellent spatial memory (Garstang 2015, 20–24). An informant in Kwandu Conservancy reported: “that is why we see corridors, these corridors have been made by ancestral elephants, they used them again and again; as long as a family does not get finished, this knowledge about the corridor [*ndjira ye zipau*] is carried on to other elephants.”^1^ The individual experience of leading animals, that is, the transgenerational spread of information but also the spread of information amongst co-living beings, may be as instrumental as visual or olfactory information. Unfortunately, we know little about the extent to which paths produced by elephants are instrumental for other types of wildlife. Rutina (2004) presents data that shows that impalas in the bordering Chobe National Park (NP) make frequent use of and benefit from elephant paths. Biological research tends to be focused on single species, and contributions that discuss corridors with a perspective that considers many species are still rare (but see Brennan et al. 2020).

The entire KAZA TFCA is framed as a coexistence landscape (Kavango Zambezi 2022). The concept of coexistence landscapes has become prominent in recent conservationist debates, signalling the turn from a strict protected-area policy (wildlife conservation can only happen in territorially bounded protected areas without extractive human land use and without human settlement) to a coexistence policy (wildlife and humans cohabit and co-adapt in a jointly used landscape in which corridors connect core conservation areas). Typically, the coexistence of humans and wildlife brings about conflict, and much of the literature on the human land-use/ wildlife interface has described and analysed such conflict in detail (König et al. 2020, 787; Fabiano et al. 2023; Whande 2023). In contrast, literature on coexistence landscapes has stressed mutual adaptation over conflict and looks at human-wildlife interaction beyond conflict.

## The emergence of wildlife corridors in north-eastern Namibia’s conservation landscape

When asked about wildlife corridors in the Mudumu Landscape, people give two answers that seem contradictory at first. Some people say that wildlife corridors are recent and that it was conservancies that set them up as part of their zonation plans. Others say that wildlife corridors have always been there and that their ancestors were well accustomed to stretches of land that were seasonally characterised by a high degree of wildlife mobility. Whilst the latter refer to wildlife migratory routes and paths trodden specifically by elephants, the former refer to the recent inauguration and demarcation of formal wildlife corridors. I begin with discussing the formalisation of wildlife corridors in the 2010s.

**Figure 1.**
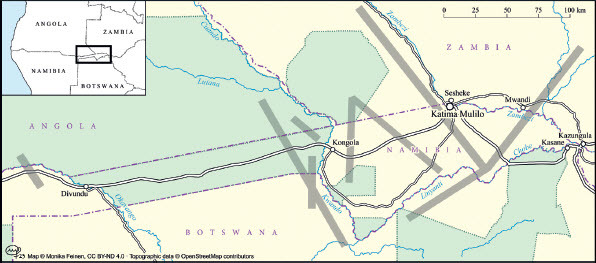
Primary wildlife migratory routes (marked by wide grey lines) running along or through the Mudumu Landscape. © Monika Feinen, reproduced by Creative Commons Licence BY-ND 4.0. Source: Based on Naidoo et al., “Evaluating the Effectiveness of Local- and Regional-Scale Wildlife Corridors using Quantitative Metrics of Functional Connectivity,” 2018.

Conservancies were inaugurated as community-based conservation organisations in Namibia’s Zambezi Region from the late 1990s onwards. To date, some 18 conservancies have been gazetted by the Ministry of Environment, Forestry and Tourism (MEFT). Most conservancies in the Zambezi Region border one of the three smaller national parks (Bwabwata, Mudumu and Nkasa Rupara) and are depicted as buffer zones, as, for example, in the Zambezi Integrated Land Use Plan. They cover about 4 100 km^2^ of rural lands in the Zambezi Region and involve c. 33 000 people as members, about 30% of the Zambezi Region’s rural population (NACSO n.d.). Original conservancy planning focused on core conservation areas, restricted areas that rural communities desisted from using. Each conservancy developed a zonation plan that demarcated such core conservation zones and differentiated them from zones for tourism, trophy hunting, farming, cattle husbandry or settlement. Whilst these zonation plans were not legally binding, they conveyed a plan for the use of a landscape. Habitually, such zonation plans were developed by conservancy committees in collaboration with experts from conservationist NGOs, were then discussed with traditional authorities and finally agreed upon in larger community meetings. Only since the early 2010s did wildlife corridors become an essential characteristic of conservation planning in northern Namibia. The focus on wildlife corridors corresponded to the international concern about connectivity and biodiversity and was linked to the agenda of the KAZA TFCA, of which the Mudumu Landscape became an essential part. In the early 2010s, maps were developed that showed prime elephant migratory routes that connected lands densely occupied by elephants in northern Botswana, the Okavango Delta, Ngamiland and the Chobe NP with the national parks in north-eastern Namibia, south-western Zambia and south-eastern Angola where elephants had been decimated during the past decades. Chase and Griffin (2009) show that a major elephant migratory route, for example, led from Chobe and Ngamiland along the western banks of the Kwandu River. The route then bifurcated. Whilst large numbers of elephants continued straight north into Angola/ Zambia, others moved through Mudumu NP into the Zambezi Region’s State Forest and then on to Zambia’s Sioma Ngwezi NP. A veterinary fence running along the northern border of Botswana separating elephant-rich Ngamiland (on the Botswanan side) from Namibia’s Bwabwata Park was removed on a stretch of about 30 km in 2012. This prime elephant migratory route has been confirmed and mapped repeatedly in scientific reports. Telemetry data from collared elephants aptly demonstrated the continued existence of this prime elephant corridor (Naidoo et al. 2018; Brennan et al. 2020). Other primary routes of wildlife migration were identified and other types of migratory wildlife such as buffalo and zebra came into the picture (Brennan et al. 2020; Taylor 2022). The map in Figure 1 depicts such primary migratory corridors; it is a simplified version of a number of maps in Naidoo et al. (2018) showing the spatial behaviour of single collared animals.

**Figure 2.**
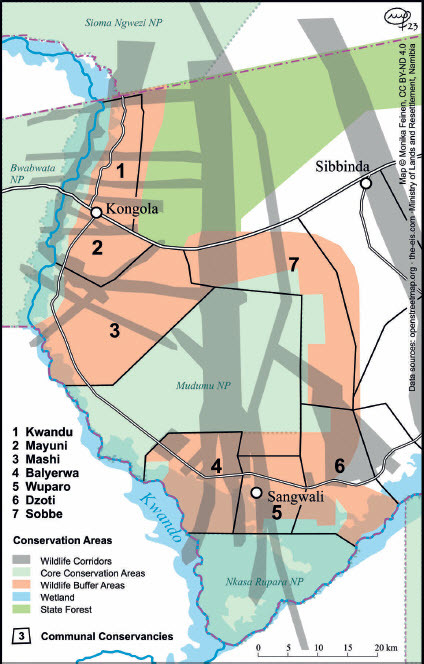
Conservancies, national parks, buffer zones and wildlife corridors in the Mudumu Landscape according to the Zambezi Land Use Plan of 2015. © Monika Feinen, reproduced by Creative Commons Licence BY-ND 4.0.

These primary routes, however, bypassed or only straddled the conservancies of the Mudumu Landscape (with the exception of the Sobbe wildlife corridor, which was indeed part of the major “highway”). In their effort to facilitate wildlife mobility, conservancies laid out wildlife corridors in considerable numbers. The empirical basis here, however, differed. Whilst the primary wildlife corridors were based on telemetric data and several aerial surveys, the secondary corridors were mainly mapped based on oral evidence and direct observation by the conservancies’ game guards. Mapping of secondary corridors started in the early 2010s, but the Integrated Land Use Plan for Zambezi Region became the first official exercise mapping these corridors for the Mudumu Landscape (MLR 2015a). The map in Figure 2 shows the corridors running through the Mudumu Landscape according to the land-use plan.

The regional land-use plan was carefully prepared in a number of meetings, with several workshops taking place in Windhoek, Katima Mulilo and respective constituencies in the region during 2014. Especially in the workshops at community level, various land-use practices were discussed and local knowledge of wildlife mobility was gathered systematically. It was combined with GIS capacity. Game guards patrolled along such corridors and entered GPS points wherever they found wildlife or saw abundant spoor. Hence, the corridors that were finally endorsed were deduced from diverse data sets and ample oral information from locals.^2^

The Zambezi Integrated Land Use Plan identified linear development along roads as a major challenge and pointed to infrastructural development (for example, the provision of a water supply via a pipeline along the major tarred road) as a key concern in this respect. Core conservation zones, buffer zones and wildlife corridors were proposed and mapped out. Buffer zones were added around the national parks to protect humans from wildlife and vice versa in a zone of 10–15 km around the parks. The planning map shows that these buffer zones comprise almost the whole of Mudumu Landscape (see MLR 2015b, 47). They are criss-crossed by numerous corridors. Three types of corridors are identified and mapped: (a) elephant corridors (from telemetry data); (b) corridors identified by communities (lumping together elephant mobility and the mobility of other wildlife); and (c) pinch points crossing major roads (again taking into account all wildlife movements). Corridors were identified through participatory community workshops, GIS data from prior conservancy zonation planning and GIS data from elephant telemetry. Figure 2 shows the corridors as laid out in the Integrated Land Use Plan (MLR 2015a, 50–52). The map also shows that many wildlife corridors cross the Mudumu Landscape and that such corridors occupy a major percentage of the overall landscape.

**Figure 3.**
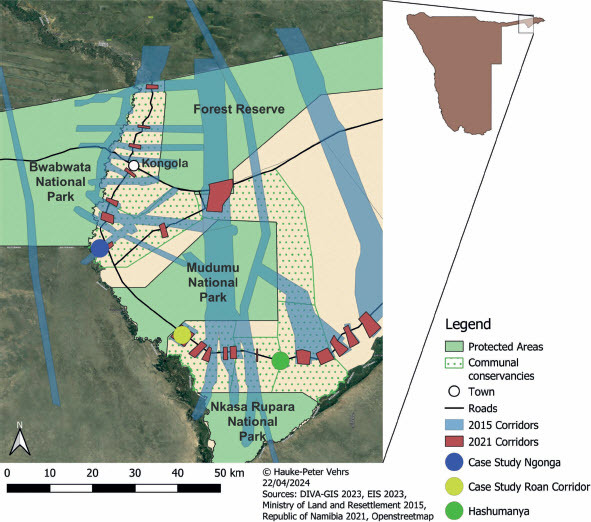
Wildlife corridors of the land-use plan and of later conservancy planning. © Hauke-Peter Vehrs

The wildlife corridors as depicted early on apparently never became the basis of immediate planning. In their day-to-day activities, conservancies and NGO staff habitually worked with the more modest pinch-point model, that is, they took one type of corridor outlined in the land-use plan as their point of departure. Hence, efforts were focused on demarcating and protecting pinch points where wildlife crossed roads, and less energy was invested in sustaining the corridors along their whole length. The map in Figure 3 shows that this led to some ambivalence because pinch points — as used, for example, in corridor planning of the conservancies and the Integrated Rural Development and Nature Conservation NGO — do not neatly correspond to the wildlife corridors in the land-use plan. Whereas the land-use plan depicts corridors as linear planning elements running through the entire landscape, the pinch points are just markers along the road. The pinch points of corridors were marked by plates first during the late 2010s and then again, with a more modern design, in early 2023.

In the following, I present sketches of three such corridors: the Hashumanya Corridor of Wuparo Conservancy, the Roan Corridor of Balyerwa Conservancy and the Ngonga Corridor of Mashi Conservancy. The Hashumanya Corridor was deemed the most important corridor of Wuparo Conservancy. It is not on the land-use plan map of 2015 but is part of the planning by the Wuparo Conservancy. Roan Corridor of Balyerwa and Ngonga Corridor of Mashi are planning elements in both land-use plans.

### The Hashumanya Corridor of Wuparo Conservancy

The Hashumanya Corridor of Wuparo Conservancy connects the Nkasa Rupara and Mudumu NPs. The Hashumanya Corridor has a width of only 200 m at the pinch point but, as it is bordered by another corridor of neighbouring Dzoti Conservancy running in the same direction, its width may reach 800 to 1 000 m (see Figure 3). The corridor is used by elephants, sable, roan, zebra and buffalo seasonally. Game guards and local farmers reported that animals use the corridor mainly at night and that this was a change from earlier times when wildlife was also seen moving along during the daytime. Farmers cultivating on or next to the corridor reported significant crop raiding by elephants in March 2023.

The corridor stretches across land that is under the control of the traditional authority of Samuduno village, and the five or six farms encroaching upon the corridor are all owned by farmers living in this village. There are a few farms at the riverside where the corridor enters Nkasa Rupara NP, but apparently these farms are not consistently used and they are spread widely apart so that wildlife can easily pass. Where the corridor crosses the road, there are some settlements, and one large homestead is right at the pinch point of the corridor at the road side. The village is old, though, and predates the formal endorsement of the corridor. Another large field of more recent origin extends into the corridor. Further onwards towards Mudumu NP, there are the farms of three brothers and their paternal relatives. At least six of these farms are placed on Hashumanya Corridor. All field owners obtained customary land titles for their farms in recent years, indicating that the land board endorsing customary land titles took little notice of the existence of the wildlife corridor. Many of these fields are no older than 10 years, pointing to a rapid encroachment on the corridor, according to a game guard accompanying our survey of the corridor. In interviews with the farmers owning these fields, however, they pointed out that their ancestors had already occasionally ploughed fields in that place, and they legitimised the placement of their fields with reference to this ancestral land use. They admitted, though, that their cattle herds stayed out there in their “bush village” all year round since they could rely on a newly built borehole there. They also asserted that the size of their farms was new. Where their ancestors had cultivated only a few hectares of land at most, the land they cultivate now amounted to several dozens of hectares. Most fields were de-bushed and de-rooted, facilitating agricultural activities. The fields were ploughed preferably by tractor or, if need be, by two- or three-oxen ploughing teams at the same time. They currently engaged about 20 to 30 Zambian farmhands who looked after the cattle, cleared the bush for farmland where necessary, did the ploughing (if it was not done by tractor), weeded and assisted with the harvest. When visiting the farms out there, we found that none of them was fenced and the efforts to keep away elephants were moderate: wires with tins were strung from tree to tree so that elephants walking into them would become irritated by the noise. All people we interviewed, however, judged this measure as ineffective but were at a loss to find other affordable means to keep away elephants and other wildlife. When interviewed, the farmers suggested that elephant mobility was not massively disturbed by the fields. They saw widespread elephant depredation of fields as strong evidence backing up their assumption that their fields did not interrupt wildlife mobility in any significant way.

### The Roan Corridor of Balyerwa Conservancy

It was in Balyerwa Conservancy that we heard the most sceptical voices about the corridors. Balyerwa Conservancy has six corridors altogether, all of them threatened by encroachment. Several officers from the conservancy but also members of the conservancy opinioned that wildlife corridors, although endorsed only recently by the conservancy, were rapidly being lost due to heavy settlement and swift expansion of agricultural activities. Balyerwa Conservancy is home to a fair number of prominent and successful community members, highly decorated politicians, lawyers, medical doctors, headmasters, teachers, public service officers and so forth. A very rough count showed that perhaps 20 to 25 people there can be categorised as wealthy. A good number of farms in Balyerwa Conservancy have 25 ha or more of land, and many large farms are indeed right on or straddling wildlife corridors. Another observation about changing settlement patterns in relation to wildlife corridors is pertinent in this context. Balyerwa Conservancy has four major villages: Lianshulu, Sauzuo, Mbambazi and Nongozi. All four villages are about 2–3 km south of the tarred road. In recent years, these villages disintegrated. People left the village to settle along the tarred road as this offered ample water (through a pipeline that was laid out along the road in 2012 when that road was tarred) and even in the bush north of the road, where some sizeable farms with buildings were set up. One former conservancy official stressed that this was not so much because of conflict but rather because of changing lifestyles and aspirations. There is a tripartite settlement structure emerging: the former key villages, marked linear settlements along the road and dispersed homesteads in the wider area. This new form of dispersed settlement is consuming much more space than the prior settlement pattern that concentrated the population in a few villages.

The Roan Corridor is Balyerwa’s biggest and also, as the conservancy’s game guards say, its most important corridor. All issues listed above pertain here as well, but community strife makes the situation of this corridor more precarious. The Roan Corridor stretches along the southern boundary of Mudumu NP. Today there is heavy settlement on this corridor. We counted about 90 households having fields and homesteads on the corridor. Most households claim that they only came to the corridor after being “ousted” from Lianshulu village because of political strife. They have to go far back in history to explain their predicament. In 1980, the administration of the East Caprivi, dominated by South African administrators, decided to forcefully relocate about 300 people from Old Lianshulu and the wetlands that were to become part of Mudumu NP in 1990 (Vehrs and Zickel 2023). They were located in a new place about 15 km away from their old homes, a place that came to be known as New Lianshulu. However, the land of New Lianshulu had been occupied by households of another family. For a couple of decades, though, all three families settled in New Lianshulu without major issues.

The implementation phase of the 2003 Communal Land Reform changed the conditions as it created the opportunity to claim customary land titles. The original landowners of New Lianshulu now opposed any claim by those who had been pushed there by the colonial administration applying for such land titles. Indeed, as the newcomers could not prove that their ancestors had ploughed these lands, they were disadvantaged when applying for land titles. Provocatively, they were told to go back to where they had come from, to the Mudumu Park area. Indeed, the descendants of those who had been forcefully relocated in 1980 made a desperate effort to regain access to the park in 2014. They protested in front of Mudumu Park’s southern gate and demanded to be allowed to return to their ancestral land. To no avail; the government turned a blind eye to their protests and told them that they should use the formal options given in Namibian law to launch such protests — an effort that resulted in no tangible results. In New Lianshulu the conflict escalated, and the old-timers told the newcomers to leave. The latter grudgingly agreed and went for the yet unsettled land that was earmarked as the Roan Corridor. Unlike some other corridors in Balyerwa, which had attracted the settlement of wealthy farmers, the farms on the Roan Corridor are small. The conflict went on. In an effort to bring the traditional Bayei authority onto their side, the erstwhile refugees applied to him for support. The traditional authority, however, sided with the original landowners. This motivated the disenfranchised community to seek support from a competing traditional authority, which finally accepted their bid. Infuriated, the Bayei authority ordered all homesteads settling on the corridor to leave the place immediately and for good and to settle with their newly selected traditional authority about 60 km away. The issue has since gone to court and is awaiting a verdict. The affected are adamant, though, that they will not leave the Roan Corridor unless they are given back ancestral land inside the park.

### The Ngonga Corridor of Mashi Conservancy

Mashi Conservancy, endowed with at least six lodges and/or camping grounds and a sizeable trophy-hunting quota, is one of the income-richest conservancies in Namibia, with money accruing from trophy hunting and joint-venture contracts with touristic enterprises. Its wildlife corridors are deemed essential to facilitate connectivity between Bwabwata NP, Mudumu NP and the State Forest. Some of its corridors also feed directly into the large Sobbe Corridor that is essential for major elephant mobility between Mudumu NP and the State Forest and connects the Botswanan corridors with Zambia’s Sioma Ngwezi NP. The corridor is today blocked by a large fence in a crop field right at the road side and some more clearings in the surrounding area, but beyond these farming activities there is no settlement or agricultural land use of the corridor for about 20 km between the village of Ngonga (which gave the corridor its name) and Lubuta village. Near Lubuta there are some more farms on the corridor. The community game guard we interviewed emphasised that the farming activities at Ngonga close to the tarred road were deplorable but that they did not impede wildlife mobility significantly. He emphasised that wildlife numbers had even increased over the past years — a fact that is well documented in publicly accessible data on wildlife demographics in north-eastern Namibia. There are sizeable eland herds crossing through Ngonga or on their way to Sobbe Corridor and the State Forest, and the number of elephants making use of the corridor is also on the increase.

Whilst the impact of the few farms on the corridor are perhaps not grave, their presence is much disputed. The traditional authority, a senior retired state official, is the owner of the main field in Ngonga and obtained a customary land title for this stretch of land. In an interview, he voiced his hope to convince an investor to assist him with utilising the field to its full extent. His status as traditional authority was highly disputed, though. He inherited his position from an outgoing sub-induna who felt too old to do the job properly. But as soon as the retired state official had taken over the headmanship, he changed his affiliation to a different senior chief, by whom he felt better supported. He argued that his part of Mashi Conservancy was now under a new chief. This, of course, brought about a storm of protest. A headman had no authority to simply break away part of the conservancy and take it to another chief. The Ngonga Corridor’s fate thus became the subject of competitive and hostile politics between two competing traditional authorities. The situation escalated in mid-2023 when local supporters of the new headman uprooted all pointers to the conservancy on “their” territory and protested in the street claiming that they should have free choice regarding by which chief they would like to be governed.

## Contested corridors

The three case studies presented above underline that wildlife corridors in the Mudumu Landscape are contested for different reasons. I briefly elaborate on three aspects here.

### Increasing numbers of wildlife

There is ample evidence that wildlife numbers increased significantly over the last three decades. Whilst Mendelsohn, Roberts and Hines (1997, 30–32) delineated a decline in some species (for example, lechwe) and mentioned the local extinction of giraffe, black rhino and wildebeest in the mid-1990s, since then the numbers of most (if not all) wildlife have increased. Mendelsohn, Roberts and Hines already reported that elephant numbers in the region were on the increase and estimated the population at about 5 000 to 6 000 elephants. The Zambezi Integrated Land Use Plan shows increasing numbers of elephant, impala (steep increase), sable, zebra and elephant in the Zambezi Region for the period 2006–2013 (MLR 2015a, 47). The number of elephants for the year 2013 is estimated at 8 401. The Elephant Status Report for Africa 2016 gives 13 116 elephants for the entire Zambezi Region (Thouless et al. 2016, 172). The website of the Namibian Conservancy Support Organisation (NACSO) gives data on elephants for smaller spatial units: whilst the two national parks Mudumu and Nkasa Rupara hold 4 598 and 2 151 elephants respectively, the number of elephants for Mudumu North is counted at 467 and for Mudumu South at 446 (that is, a total of 7 662 elephants for the entire Mudumu Landscape). Other animal species increased their populations as well. The Corridor Report gives an increase in impala numbers from 12 676 (2013) to 14 623 (2019) and in hippopotamus numbers from 821 (2009) to about 3 000 (2019) (GoN 2022). Whilst some wildlife species are still endangered, like the puku with a mere 15 individuals or the African wild dog with about 70 individuals according to the same report, there is no doubt that, on the whole, wildlife numbers increased — just as cattle numbers did.

**Figure 4.**
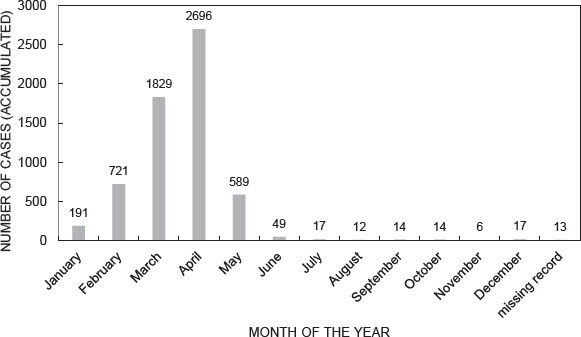
Reported cases of human-wildlife conflict involving elephants in Mudumu North and Mudumu South between 2004 and 2020 (Note: The figure is based on the analysis of reported human-wildlife conflict cases recorded by conservancy game guards in the seven conservancies of the Mudumu Landscape. The dataset has been cordially offered by NACSO).

Increasing wildlife numbers lead to more conflict but also to more intimacy and knowledge. Data recorded by community game guards and made accessible by NACSO show a distinct pattern in elephant crop raiding in the Mudumu Landscape for the years 2004 to 2020 (Figure 4). Elephant raids were recorded in exceedingly high numbers in the months of February, March and April when the crops on the fields were close to harvest. During my fieldwork in March and April 2023, many people claimed that they were on their fields every night, safeguarding them against elephants, buffalo and hippos. They claimed that elephants raid crop fields in a targeted and concerted manner. Occasionally staff of the Ministry of Environment, Forestry and Tourism also came to assist farmers and some shots were fired into the air to frighten elephants. Often all efforts were in vain, and the damage to crop fields was substantial. Figure 4 lists 6 026 cases of crop raiding by elephants over 16 years for the Mudumu Landscape; that is about 380 cases of crop raiding per year. During the key harvest months, elephants regularly raided maize fields.

Increasing human-elephant interaction was closely linked to profound and in-depth knowledge of elephant sensing, sociality and memory. Local people were unanimous in their opinion that more knowledge of elephants would help to contain conflict to some degree (see also Mumby and Plotnik 2018). In a number of open-ended interviews, informants stressed the superior capacity of elephants’ smelling senses. They alleged that elephants could smell objects and also humans over large distances and could communicate a smell once captured with their trunk to fellow elephants.

They also theorised that smell would trigger other senses, that is, that a distinct smell would motivate elephants to capture an object visually. Elephants displayed complex social behaviour with intricate authority structures, patterns of socialisation and conflict. Elephants were fitted with an immense memory that allowed them to connect places to specific experiences that were many years past. Local informants were adamant that elephants adjust their behaviour and their mobility. They evaded dense human settlements, seeking different routes and resorting to nocturnal mobility to evade humans. Elephants remembered places where they had found food just as much as places where they had been shot at and one of them had died. (This conforms to ethological literature on elephant memory. Garstang [2015, 100] speaks of a “total neural memory of the sensory environment of their home territory.”) Informants assumed that, because of their specific history, Namibian elephants behaved differently than Botswanan elephants. They alleged that Namibian elephants learnt well to coexist with humans whilst much of the intense human-elephant conflict resulted from Botswanan elephants that were less well adapted. Wildlife, of course, is not only concentrated in wildlife corridors, and some species are not at all attached to corridors but roam about. Nevertheless, these figures indicate that cattle and wildlife alike increasingly use natural resources within corridors. The increase in numbers of diverse wildlife species brings about a rearrangement of interrelations, competitive and symbiotic. There is as yet little research that looks at changing interspecies relations in such a set-up where different species increase their populations at the same time. Cattle compete with some species (for example, zebra) but not with others (such as impala). Zoonotic diseases can jump from buffalo to cattle, and some cattle diseases may affect other ungulates. It is the conjunction of increased wildlife/livestock impact, rearrangements of interspecies relations and increased human land use that is significantly shaping corridors.

### Communal land reform, customary land titles and the expansion of farmlands

The Communal Land Reform of 2003 took about 10 years to be fully rolled out in the communal areas of the Zambezi Region. It took time to set up a land board in the region that was to govern communal land reform. Plots of up to 25 ha (today up to 50 ha) could be registered, and an individual was entitled to register five such plots. The registration of communal land rights was advertised through radio, adverts and by word-of-mouth propaganda. It was especially the educated and those with ample monetary funds at their disposal who went for land titles. They quickly saw the benefits of such a registration. These are often part-time farmers, who earn (or have earned) their incomes as civil servants, lawyers, medical doctors and politicians in high-ranking governmental functions. Many part-time farmers take a good share of their retirement money (of which one part can be paid out as a lump sum when the employee reaches the age of 60) to establish large farms, drill boreholes or fence lands. Their agricultural projects are certainly based on an economic calculus. At the same time, however, they are ways to express individual success in the local arena that serve to reintegrate these successful labour migrants in the rural society. Large herds of cattle and expansive fields make their owners members of a rural society, express values of rurality and signal their attachment to local traditional authorities. The surveyed and registered boundaries would create security vis-á-vis neighbours and within larger families. Whereas previously land was held by patrilineal families, now lands could be registered in the name of a single person. The option to register major portions of land created new options for wealthy people. They would have funds at their disposal to clear major plots for ploughing and cultivation, to drill private boreholes and fence in large areas of land. They also had the capacity to employ a larger number of farm workers to cut back the forest, clear the ground of roots, plough, sow, weed and harvest.

Interviews underlined that a rapid process of land accumulation is taking place and that especially amongst the literate there is a real run for land titles. One informant put it succinctly:

The literate group sees advantage in a certificate so that even if I am a member of a family [that has land], I go and get a certificate and take major pieces of land to myself. If you find somebody having 100 ha of land that is not ordinary land, he has grabbed land from others. Others are now without land because he has taken their land.^3^

**Figure 5.**
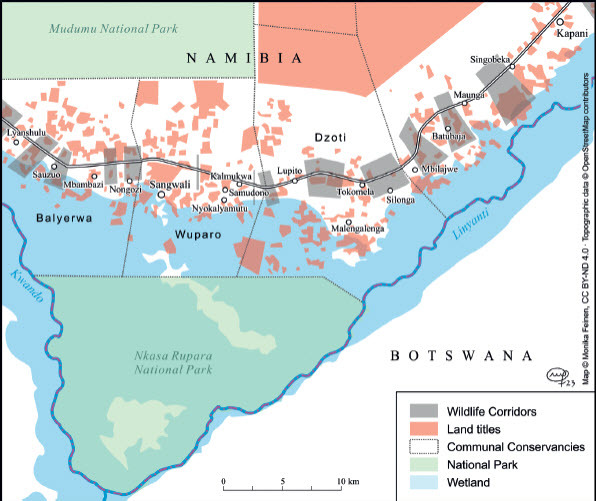
Conservancies with corridors and customary land titles given until 2019. Source: Based on a map produced and cordially granted access to by Katharina Dierkes, World Wide Fund for Nature, Windhoek, 2020. © Monika Feinen, reproduced by Creative Commons Licence BY-ND 4.0.

The map in Figure 5 shows the registration process for customary land titles in Mudumu South with land titling data up to March 2020. The map shows that many wildlife corridors are today blocked by farms, in Balyerwa and Wuparo Conservancies even intensively so.

As long as there are no fences, the farms do not directly impede wildlife movement; in fact, they may unwantedly contribute to it. As pointed out above, elephant depredation of crop farms is an issue of enormous significance.

Crop cultivation on and near elephant corridors both attracts elephants during the months February to May and hampers their mobility because of human presence. Human-elephant interaction is intense over these months of the year. Many farmers bemoan that they have to spend night after night on their fields to fend off elephants. I talked to many enervated and exhausted farmers who had fended off elephants for weeks in a row, some of them in vain as elephants had finally won the battle for the crops. They claimed that these elephants were now used to humans; they were no longer shy and without doubt clever enough to know that farmers could make a lot of noise but would certainly not have the capacity to harm them. Humans were also sure that elephants communicated their findings about promising crop fields to their fellows and thereby the entire elephant population learnt how to gain benefit from altered farming practices. One informant reported:

Our expanding fields are feeding the elephants these days. Elephants are no longer shy. In the past people used to beat drums and the elephants would run away. They used a whip, and it sounded like a rifle. The elephants would run away. But today you can make any noise and the elephant will just stand and wait until the man or woman gets tired and then they will get into the field. They are clever animals.^4^

### Changing settlement patterns and political strife

The competitive run for land is accompanied by changing settlement patterns. Two developments are of significance in this respect: (1) the gradual dissolution of major villages and (2) linear settlement along major roads. I deal with both processes separately, although they are linked. Villages have been large and consolidated in the past, with distinct boundaries and a high population density within the perimeter of the village. Schools, health clinics and the presence of the traditional authority in the village guaranteed a high degree of settlement stability. (In the more distant, precolonial past, settlement was apparently much more dispersed [Bollig and Vehrs 2020], a fact that I cannot expand upon here.) In recent years, more and more people prefer to settle beyond the limits of the village. Wealthy people nowadays often build spacious brick houses in a radius of 2 to 3 km around a village and/or close to the tarred road. In other places, other mechanisms drive the dissolution of the village.

Increasingly, people settle close to the road that was tarred in 2012. All transport passes along this road that connects the villages of the Mudumu Landscape to the region’s capital, Katima Mulilo. Therefore, if transport is needed or goods are to be transported, having one’s homestead close to the road pays off. When the road was tarred, the Directorate of Water Affairs laid down a pipeline along the road. People settling close to the road can apply for a pipe and a metre, which then brings water directly to their homestead. Over the past 10 years, many households have relocated from the major villages, which tend to be 2–5 km away from today’s tar road, to places directly at the roadside. Conservationists bemoan this rapid increase in linear settlement. They point out that it is not so much the road or the traffic but the dense linear development along the road that is hampering wildlife mobility. A coordinator for wildlife corridors from a leading NGO reported of a talk he had with the head of operations for Namwater in Zambezi Region, the agency responsible for the supply of water. The coordinator informed the head of operations that the deployment of water pipelines along the road was detrimental to wildlife corridors and pleaded with him not to consent to applications for installing water metres in cases where they discovered that such a homestead was within a wildlife corridor. The responsible person, however, complained that he is only implementing a government programme that promises clean water to all citizens.

Political strife within communities, often linked to the competition between traditional authorities for followers, lands under their control and income, was essential in two of the three cases accounted for here. With reference to the past, communities formulate claims to land, and they actively try to obstruct applications for land rights of those whom they feel are not entitled to do so because of late immigration or affiliation to another chief. Often conflicting families seek support from competing traditional authorities. Typically, such conflicts over access to land are brought to court and it takes years to resolve them. In such a situation of severe internal political strife, conservancy committees are sidelined. Their zonation plans are invalidated when traditional authorities do not support them. Hence, many argue that the challenges of Roan or Ngonga Corridors cannot be solved before the traditional authorities find an amicable settlement of conflicting claims.

## In search of solutions: projects, programmes, ideas

There is currently a run for solutions for the conundrum of wildlife corridors: how can they be maintained without compromising the economic prospects of local farmers and risking their food security?

Many local conservationists allege that only if there is a sizable profit accruing from a corridor will people be motivated to keep up the functioning of the corridor and desist from using it for other purposes. Also, locals suggest that they ought to benefit directly from the corridor because they own the land on which the corridor runs and they face opportunity costs from wildlife damage. Hence, there is considerable agreement that a wildlife corridor must be valorised, perhaps to the point that it must become a commodity in order to be working — a commodity that a local community can trade in if it can prove that its ecosystem functions are well-maintained. Greiner and Bollig (2023) spell out conditions for the commodification of natural resources. Who can be an acknowledged owner or manager of a stretch of land that formally is owned by the state and since decades has been administered by traditional authorities together with all other community land? One answer is that a wildlife corridor is an essential part of a conservancy’s zonation plan. Hence, the conservancy is the legitimate manager of it on behalf of the state. Others claim that only those people who have ancestral land rights pertaining to the corridor should benefit and manage such lands or, in another version, only these who hold customary land titles in the corridor. A third group argues that the traditional authority is the real owner of empty stretches of land and corridors should first of all be managed by them. Yet others seek for a compromise and suggest that those with ancestral land rights should be prioritised but that a certain percentage of gains accruing from a corridor should go to the conservancy and the traditional authority.

Several legal options are discussed as to how to formally endorse corridors. Could a wildlife corridor become a formal leasehold owned by the conservancy? Alternatively, could occupational land rights be obtained for a wildlife corridor? Like a school needs a large compound, a conservancy needs wildlife corridors for its existence. Communities are ambitiously discussing whether corridors can be alienated. Castree (2003, 279–280) presents alienability as a key feature of commodification processes and defines the concept as the “capacity of a given commodity … to be physically and morally separated from their sellers.” This is exactly where discussions in the region are at the moment: on account of what reasoning can corridors be alienated and, if so, how can they be physically defined and monitored in an effective way? Abstraction — defined as a “process whereby the qualitative specificity of any individualized thing is … assimilated to the qualitative homogeneity of a broader type or process” (Castree 2003, 281) — is another essential element of commodification. Indeed, the process of defining wildlife corridors in the region necessitates such homogenisation and an approach to define and handle corridors in a similar manner.

The recent Wildlife Credits Project, pushed by the Namibia office of the World Wide Fund for Nature, has endeavoured to find a solution to these complex and controversial debates. The project is based on funding that a major liquor company offered to reward communities that actively protect and conserve wildlife corridors. Facilitated through the Wildlife Credits Project, the company donated a one-time sum of NAD 130 000 for the Sobbe Conservancy of Mudumu North, remunerating it for its successful stewardship of the corridor. The linkage between wildlife credits and wildlife corridor maintenance is still at an experimental stage, though. Neither is it easy to define the group of wildlife corridor stewards (the conservationist perspective) or owners (the local perspective) clearly, nor is it without problems how to define a well-kept corridor.

Whilst the commodification of a corridor demands intense social labour, engaging politicians, lawmakers, conservationists and conservancy members in discussions, others opt for more technical solutions involving improved monitoring along those wildlife corridors that allow for more surveillance of wildlife. Indeed, the previous years have seen a number of technology-based smart solutions to monitor wildlife mobility in a better way. Game guards patrol corridors at least once per month on foot and record all wildlife sightings and spoor along the way. Thereby they provide an excellent overview of wildlife activity. Recently game guards were fitted with smartphones with an app that helps to record wildlife sightings, spoor and other incidents worth mentioning. The information is directly communicated to NACSO headquarters in Windhoek. However, critics assert that, whilst this produces more knowledge of wildlife activity and helps assess wildlife mobility along the corridor, it neither addresses the crucial problems that wildlife corridors are facing nor helps to preclude human-wildlife conflict.

## Conclusion

To look at wildlife corridors as infrastructure co-produced by humans and wildlife adds to the understanding of human-wildlife interaction in conservation landscapes. The infrastructure perspective suggests that corridors are the result of negotiations between people equipped differently with power and interaction between people and wildlife. Whilst major corridors in the KAZA conservation landscape may have a long history, the corridors dealt with in this contribution were the result of changing settlement patterns, intricate planning and grassroots-level discussions and disputes, on the one hand, and elephant behaviours and adaptations, on the other.

This article also shows that wildlife corridors in Namibia’s Mudumu conservation landscape are in dire straits, as both conservationists and members of local conservancies claim. They warn that, if the government does not intervene, corridors will die and the entire KAZA project, which is all about the connectivity of wildlife populations and community-based organisation, is endangered. Others hold that wildlife numbers are higher than ever (within the past 50 years) and refer to the increase in, for example, elephant numbers. Yet others claim that the lands in question are farming lands that have been owned by local families for generations. Many informants stressed that there is little land without an owner and that conservancies cannot easily claim such lands for wildlife corridors. Planning for wildlife corridors needs to be better coordinated with rural livelihoods and contemporary farming and settlement practices and, specifically, the aspirations of emergent commercial farmers. Conflicts between traditional authorities and within communities need to be considered. Too often the continuous use of the concept “community” conveniently glances over the fact that settlements are characterised by severe conflict over land and a competition between traditional authorities for influence. The case studies underline that what outsiders see as a “community” is in fact a politically fragmented population that cannot easily arrive at decisions over land.

These human dimensions are multiple, as the discussion above has shown, and personal aspirations need to be considered too, beyond the political considerations. Certainly, many argue that corridors should yield more benefits to the owners of the land the corridor is running through, or alternatively to the conservancy of which the corridor is part (which are two altogether different things, of course). Yet others give a clear priority to agricultural expansion. They assert that a growing population needs more farmland or, alternatively, argue that only large crop farms can be commercially successful. They do not want to see their crop farms compromised by wildlife corridors. Hence, more benefits accruing to the conservancy may not easily solve this dynamic situation. Further, issues of how to exactly value an intact wildlife corridor and how to establish whether a corridor is intact raises further intricate questions. During local meetings, requests for remuneration that amounted to millions of Namibian dollars were voiced. Whilst this seems ridiculous at first sight, it shows that it is not at all easy to attach value to such kind of an infrastructure. It is also discussed intensely whether a wildlife corridor is only fully functioning when there is no human land use at all on that stretch of land. Or can wildlife tolerate a few farms that are active for a few months a year, if at all, on the corridor?

Wildlife has apparently adapted well to changing circumstances. As pointed out, wildlife numbers are on the increase. Wild animals may have changed their exact paths, but their mobility has not ended. Further research is required into exactly how wildlife adapts to human land use and whether there are specific tipping points at which wildlife mobility is severely hampered. There is astoundingly little information on precisely how wildlife co-adapts to changing circumstances. Wildlife, and particularly elephants, seem to profit from agricultural expansion. Crop raiding by elephants is significant, and during the rainy season maize and millet may make up a major percentage of elephants’ daily food intake. Perhaps the Mudumu Landscape is already much more a coexistence landscape than generally assumed — only that coexistence is not a peaceful, harmonious staying together but a conflictual negotiation between the two sides and a sharing of resources. These conflicts between humans and wildlife, and between humans vying for influence on decisions pertaining to wildlife corridors, demand management. The acknowledgement of wildlife corridors as environmental infrastructure co-created by humans and wildlife necessitates a planning agenda, and this requires (human) responsibility. Wildlife corridors are not simply remains from prior wilderness areas; they are, and need to be, maintained as humanly endorsed and managed infrastructure.

## Notes

1 Interview, Kwandu Conservancy, September 2023.

2 Interview with V. Schiller, GIZ advisor to the compilation of the Zambezi Integrated Land Use Plan.

3 Interview with anonymous informant, March 23, 2023.

4 Interview with M.M., March 10, 2023.

## References

[CIT0001] Barua, M. 2014. “Circulating Elephants: Unpacking the Geographies of a Cosmopolitan Animal.” *Transactions of the Institute of British Geographers* 39 (4): 559–573. 10.1111/tran.12047

[CIT0002] Bollig, M., and H.-P. Vehrs. 2020. “Abundant Herds: Accumulation, Herd Management and Land-Use Patterns in a Conservation Area.” *Pastoralism* 10: 20. 10.1186/s13570-020-00175-0

[CIT0003] Bollig, M., and F. Krause. 2023. *Environmental Anthropology: Current Issues and Fields of Engagement*. Bern: Haupt Verlag.

[CIT0004] Brennan, A., P. Beytell, O. Aschenborn, P. du Preez, P. Funston, L. Hanssen, J.W. Kilian, G. Stuart-Hill, R.D. Taylor, and R. Naidoo. 2020. “Characterizing Multispecies Connectivity across a Transfrontier Conservation Landscape.” *Journal of Applied Ecology* 57 (9): 1700–1710. 10.1111/1365-2664.13716

[CIT0005] Castree, N. 2003. “Commodifying What Nature?” *Progress in Human Geography* 27 (3): 273–297. 10.1191/0309132503ph428oa

[CIT0006] Ceballos, G., P.R. Ehrlich, and R. Dirzo. 2017. “Biological Annihilation via the Ongoing Sixth Mass Extinction Signaled by Vertebrate Population Losses and Declines.” *Proceedings of the National Academy of Sciences of the United States of America* 114 (30): E6089–E6096. 10.1073/pnas.1704949114 28696295 PMC5544311

[CIT0007] Chase, M.J., and C.R. Griffin. 2009. “Elephants Caught in the Middle: Impacts of War, Fences and People on Elephant Distribution and Abundance in the Caprivi Strip, Namibia.” *African Journal of Ecology* 47 (2): 223–233. 10.1111/j.1365-2028.2008.01017.x

[CIT0008] Dittmann, J., and D. Müller-Mahn. 2023. “Transfrontier Conservation Governance, Commodification of Nature, and the New Dynamics of Sovereignty in Namibia.” In *Conservation, Markets and the Environment in Southern and Eastern Africa*, edited by M. Bollig, S. Lendelvo, A. Mosimane, and R. Nghitevelekwa, 107–134. Woodbridge: James Currey.

[CIT0009] Fabiano, E., S. Lendelvo, A. Mosimane, S. Kosmas. 2023. “Human-Wildlife Interaction, Rural Conflict, and Wildlife Conservation.” In *Conservation, Markets and the Environment in Southern and Eastern Africa*, edited by M. Bollig, S. Lendelvo, A. Mosimane, and R. Nghitevelekwa, 305–328. Woodbridge: James Currey.

[CIT0010] Fraser, C. 2009. *Rewilding the World: Dispatches from the Conservation Revolution*. New York: Picador.

[CIT0011] Garstang, M. 2015. *Elephant Sense and Sensibility: Behavior and Cognition*. London: Academic Press.

[CIT0012] GoN (Government of Namibia, Ministry of Wildlife, Environment, Tourism and Forestry). 2022. *Zambezi Wildlife Corridor Strategy*. Windhoek. Government Printers.

[CIT0013] Greiner, C., and M. Bollig. 2023. “Fetishizing the Wild: Conservation, Commodities, and Capitalism.” In *Conservation, Markets and the Environment in Southern and Eastern Africa*, edited by M. Bollig, S. Lendelvo, A. Mosimane, and R. Nghitevelekwa, 31–55. Woodbridge: James Currey.

[CIT0014] Hilty, J., W. Lidicker, and A. Merenlender. 2006. *Corridor Ecology: The Science and Practice of Linking Landscapes for Biodiversity Conservation*. Washington: Island Press.

[CIT0015] Kalvelage, L., J. Revilla Diez, and M. Bollig. 2020. “How Much Remains? Local Value Capture from Tourism in Zambezi, Namibia.” *Tourism Geographies* 24 (4–5): 759–780. 10.1080/14616688.2020.1786154

[CIT0016] Kalvelage, L., J. Revilla Diez, and M. Bollig. 2023. “Valuing Nature in Global Production Networks: Hunting Tourism and the Weight of History in Zambezi, Namibia.” *Annals of the American Association of Geographers* 113 (8): 1818–1834. 10.1080/24694452.2023.2200468

[CIT0017] Kavango Zambezi. 2022. “Promoting Coexistence of Wildlife and People.” KAZA News, February 1, 2022. https://www.kavangozambezi.org/2022/02/01/promoting-coexistence-of-wildlife-and-people/

[CIT0018] König, H., C. Kiffner, S. Kramer-Schadt, C. Fürst, O. Keuling, and A.T. Ford 2020. “Human-Wildlife Coexistence in a Changing World.” *Conservation Biology* 34 (4): 786–794. 10.1111/cobi.13513 32406977

[CIT0019] Lacan, L., H.-P. Vehrs, P. Alexiou, M. Bollmann, J. Brekl, E. Köhler, W. van Engelen, and M. Bollig. Forthcoming. “The Assemblage in Multispecies Studies: Rethinking a Framework for Anthropological Research.” *Sociologus*.

[CIT0020] Locke, P. 2013. “Explorations in Ethnoelephantology: Social, Historical, and Ecological Intersections between Asian Elephants and Humans.” *Environment and Society* 4: 79–97. 10.3167/ares.2013.040106

[CIT0021] Locke, P., and U. Muenster. 2015. “Multispecies Ethnography.” In *Oxford* *Bibliographies* *Online in Anthropology*, edited by L.D. Baker. Oxford: Oxford University Press. 10.1093/obo/9780199766567-0130

[CIT0022] Lorimer, J. 2010. “Elephants as Companion Species: The Lively Biogeographies of Asian Elephant Conservation in Sri Lanka.” *Transactions* 35 (4): 491–506. 10.1111/j.1475-5661.2010.00395.x

[CIT0023] Mayer, M., C. Hulke, J. Kamwi, H. Kolem, and J. Börner. 2022. “Spatially Heterogeneous Effects of Collective Action on Environmental Dependence in Namibia’s Zambezi Region.” *World Development* 159: 106042. 10.1016/j.worlddev.2022.106042

[CIT0024] Mendelsohn, J., C. Roberts, and C. Hines. 1997. *An Environmental Profile and Atlas of Caprivi*. Windhoek: Ministry of Environment and Tourism, Directorate of Environmental Affairs.

[CIT0025] MLR (Ministry of Lands and Resettlement). 2015a. *Baseline Report (Volume 1) for the Zambezi: Integrated Regional Land-Use Plan (Zambezi IRLUP)*. Windhoek: Government Printers.

[CIT0026] MLR (Ministry of Lands and Resettlement). 2015b. *Integrated Land Use Plan for the Zambezi Region (Volume 2) (Zambezi IRLUP)*. Windhoek: Government Printers.

[CIT0027] Mumby, H.S., and J.M. Plotnik. 2018. “Taking the Elephants’ Perspective: Remembering Elephant Behavior, Cognition and Ecology in Human-Elephant Conflict Mitigation.” *Frontiers in Ecology and Evolution* 6: 122. 10.3389/fevo.2018.00122

[CIT0028] Münster, U. 2016. “Working for the Forest: The Ambivalent Intimacies of Human-Elephant Collaboration in South Indian Wildlife Conservation.” *Ethnos* 81 (3): 425–447. 10.1080/00141844.2014.969292

[CIT0029] Naidoo, R., J.W. Kilian, P. du Preez, P. Beytell, O. Aschenborn, R.D. Taylor, and G. Stuart-Hill. 2018. “Evaluating the Effectiveness of Local- and Regional-Scale Wildlife Corridors using Quantitative Metrics of Functional Connectivity.” *Biological Conservation* 217: 96–103. 10.1016/j.biocon.2017.10.037

[CIT0030] NACSO (Namibian Association of CBNRM Support Organisations). n.d. “Registered Communal Conservancies.” Accessed March 12, 2024. https://www.nacso.org.na/conservancies

[CIT0031] Remis, M., and C.A. Jost Robinson. 2020. “Elephants, Hunters, and Others: Integrating Biological Anthropology and Multispecies Ethnography in a Conservation Zone.” *American Anthropologist* 122 (3): 459–472. 10.1111/aman.13414

[CIT0032] Rutina, L. 2004. “Impalas in an Elephant-Impacted Woodland: Browser-Driven Dynamics of the Chobe Riparian Zone, Northern Botswana.” PhD diss., Agricultural University of Norway.

[CIT0033] Stott, P., and S. Sullivan. 2000. *Political Ecology: Science, Myth and Power*. London: Arnold.

[CIT0034] Taylor, R. 2022. “Elephant and Other Wildlife Movements across KAZA.” Paper presented at the MEFT Corridors Workshop, Katima Mulilo, February 14–15, 2022.

[CIT0035] Thouless, C.R., H.T. Dublin, J.J. Blanc, D.P. Skinner, T.E. Daniel, R.D. Taylor, F. Maisels, H.L. Frederick, and P. Bouché. 2016. “African Elephant Status Report 2016: An Update from the African Elephant Database.” Occasional Paper of the IUCN Special Survival Commission No. 60. Gland: International Union for Conservation of Nature and Natural Resources.

[CIT0036] Vehrs, H.P., and M. Zickel. 2023. “Can Environmental Injustices be Addressed in Conservation? Settlement History and Conservation-Induced Displacement in the Case of Lyanshulu in the Zambezi Region, Namibia.” *Human Ecology* 51: 89–105. 10.1007/s10745-022-00383-9

[CIT0037] Whande, T. 2023. “Human, Livestock and Wildlife Interactions at the Boundary of Hwange National Park and Tsholotsho Communal Areas in Zimbabwe.” PhD diss., University of Cologne.

[CIT0038] Wilson, E.O. 2016. *Half-Earth: Our Planet’s Fight for Life*. New York: Liveright.

